# Chemical Treatment of Waste Abaca for Natural Fiber-Reinforced Geopolymer Composite

**DOI:** 10.3390/ma10060579

**Published:** 2017-05-25

**Authors:** Roy Alvin J. Malenab, Janne Pauline S. Ngo, Michael Angelo B. Promentilla

**Affiliations:** 1 Chemical Engineering Department, De La Salle University, Manila 0922, Philippines; roy.malenab@dlsu.edu.ph (R.A.J.M.); janne_ngo@dlsu.edu.ph (J.P.S.N.); 2 National Research Council of the Philippines, Taguig, Metro Manila 1631, Philippines

**Keywords:** waste utilization, abaca (Manila hemp) fiber, chemical treatment, fiber-reinforced composite, geopolymer, tensile strength, definitive screening design of experiment

## Abstract

The use of natural fibers in reinforced composites to produce eco-friendly materials is gaining more attention due to their attractive features such as low cost, low density and good mechanical properties, among others. This work thus investigates the potential of waste abaca (Manila hemp) fiber as reinforcing agent in an inorganic aluminosilicate material known as geopolymer. In this study, the waste fibers were subjected to different chemical treatments to modify the surface characteristics and to improve the adhesion with the fly ash-based geopolymer matrix. Definitive screening design of experiment was used to investigate the effect of successive chemical treatment of the fiber on its tensile strength considering the following factors: (1) NaOH pretreatment; (2) soaking time in aluminum salt solution; and (3) final pH of the slurry. The results show that the abaca fiber without alkali pretreatment, soaked for 12 h in Al_2_(SO_4_)_3_ solution and adjusted to pH 6 exhibited the highest tensile strength among the treated fibers. Test results confirmed that the chemical treatment removes the lignin, pectin and hemicellulose, as well as makes the surface rougher with the deposition of aluminum compounds. This improves the interfacial bonding between geopolymer matrix and the abaca fiber, while the geopolymer protects the treated fiber from thermal degradation.

## 1. Introduction

The growing concerns for the environment and sustainability have served as a strong drive for researches on developing eco-friendly composite materials [1]. Composite materials in general are formed by combining two or more constituent materials, a continuous medium called matrix and the dispersed phase/s, either fiber/s or particulate/s, in order to develop another material with desired combination of properties [2]. In fiber-reinforced composite (FRC) materials, such matrix is reinforced by fibers for their superior properties and load transfer characteristics. Recently, natural fibers such as cotton, flax, jute, sisal and hemp are also becoming more attractive than synthetic fibers because of their natural abundance, low cost, low density, good mechanical properties, nontoxicity, etc. [1,2,3,4]. For example, abaca fibers have been widely studied as a reinforcement in cement [5], polypropylene [6,7] and epoxy [8] matrices, among others.

Abaca fiber or Manila hemp, known for its commendable mechanical strength, has been produced and widely exported by the Philippines. Commercial grade abaca has density 1.5 g/cm^3^ and tensile strength of about 980 N/mm^2^ [9]. In 2015, the annual abaca fiber production was 70,400 metric tons supplying around 87% of world requirements [10]. These fibers are extracted from a native banana species and are harvested mostly for their use in making ropes, in textile, and more recently, in automotive [11]. The extraction processes from plant to usable fibers produce considerable amount of waste or scrap fibers, which are still underutilized [12].

This study thus explores the utilization of scrap abaca fiber as reinforcement for fly ash-based geopolymer matrix. Geopolymer is an inorganic polymer formed from the reaction of materials rich in reactive alumina and silica with an alkaline solution [13,14]. This material has also attracted widespread scientific and industrial interest for its potential to valorize waste or industrial by-product such as coal ash while producing cementitious material with performance comparable to that of Portland cement in many applications [15]. Furthermore, it has several other advantages such as superior heat and fire resistance, high chemical resistance, and lower carbon footprint and embodied energy. Accordingly, fiber-reinforced geopolymer composite could potentially provide better mechanical and thermal properties over a wide temperature range as compared to Portland cement or organic polymer-based matrix. The use of natural fibers such as bamboo, cornhusk, wool, cotton, or flax in geopolymer matrix has been mentioned [16,17,18] and shown with promising results in the recent critical review on fiber-reinforced geopolymers [19,20]. Another study also demonstrates the surface modification of such fiber which improves its alkali resistance and results to an improved flexural strength of a reinforced metakaolin-based geopolymer as compared to that of pristine geopolymer [21]. In contrast, one recent exploratory study [22] reported no significant differences in the flexural strength between that of pristine fly ash based-geopolymer and that of geopolymer reinforced with the untreated coconut coir, cotton, or sisal fibers. This suggests that surface modification of the natural fibers is essential to improve the performance of the reinforced geopolymer composites.

To our knowledge, no studies have been reported yet on a chemical treatment of abaca fiber used for reinforcing fly ash-based geopolymer. Surface modification of abaca fibers could improve the interfacial adhesion with such geopolymer matrix. In general, the matrix and fibers retain their physical and chemical identities in FRCs, forming boundaries or interfaces [23]. Effectiveness of fiber reinforcement in composites depends on the degree or mechanism of bonding at the fiber–matrix interface and the ability to transfer stress from matrix to the fiber [24]. Stress transfer among the components should be effective in order to attain desired strength. The developed interface between the fiber and the matrix is thus a crucial factor of the composite’s static and dynamic stability [2]. This interface enables load transfer between the components. When the fiber is brought in contact with the matrix, the chemical groups on the fiber surface can bond with those present on the matrix. Electrostatic, hydrogen bonds and dipole-dipole interaction can develop depending on the nature and compatibility of the fiber and matrix. This is the basis for the fiber's adhesion to the matrix. Surface treatment, which can modify or introduce chemical groups that could aid in bonding, is often applied to render the fiber compatible with the matrix. Meanwhile, the presence of hydroxyl and carboxylic groups in natural cellulosic fibers' structure make them intrinsically hydrophilic and polar. When such fibers are used as reinforcement in hydrophobic matrices, like polymers, the resulting composite is a heterogeneous system that could have inferior properties due to lack of adhesion and chemical affinity. 

Some of the organic components of natural fibers can inhibit its compatibility with the targeted organic or inorganic matrix. Since the interface quality and bondage between matrix and fiber is insufficient, surface modification of these fibers through chemical treatment and other techniques have been pursued in research for years such as alkali, chemical coupling, oxidation, plasma, ultrasound, and enzyme treatments [23,25]. The most common treatment for natural fibers discussed in literature is alkali treatment, where the fiber is soaked in sodium hydroxide (NaOH) solution in order to strip off some of the unwanted waxes, hemicellulose and lignin. Due to the hydrophilic nature of some of the fiber components and thus, tendency for water absorption, natural fibers may exhibit weak interfacial bonding with hydrophobic matrices [26,27]. Furthermore, “swelling” may occur when a hydrophilic fiber within a hydrophobic matrix absorbs moisture within the matrix, making it susceptible to weakening and deterioration [28]. This may occur in the event that water absorption due to the fiber breaks hydrogen bonds between the fiber and matrix or among the fibers themselves. In addition, alkali treatment induces rougher surfaces for better adhesion between the fiber and matrix. Prior studies have also shown that alkali treatment of these fibers have improved the performance of reinforced composites in terms of thermal stability [29], water absorption and tensile strength [30,31].

Degradation of such natural fibers when used in cementitious or geopolymer matrix due to the harsh environment is another concern. The decay of plant fibers when used as reinforcement in alkaline matrices such as that of concrete has been observed [32]. Therefore, plant-based fibers must be treated not only to be compatible to its matrix but also to avoid or delay its degradation ensuring that its reinforcing function is sustainable. Aluminum sulfate treatment of natural fibers is a potential technique to address the possible deterioration of plant-based fibers exposed to cementitious matrix. This technique has been employed for pulped wood fibers to reinforce cementitious matrices [33]. The treatment involves deposition of insoluble aluminum hydroxide on the fiber surface via neutralization process. These ionic deposits are expected to have better affinity with the inorganic or cementitious matrix when compared to the affinity of organic, hydrophilic nature of cellulosic fibers to such matrices. 

In the present work, we investigate the effect of successive chemical treatment via NaOH pretreatment followed by an aluminum sulfate treatment of waste abaca fibers on their tensile strength and morphology. Fourier transform infrared spectroscopy (FTIR), scanning electron microscopy with energy-dispersive X-ray spectroscopy (SEM-EDX), X-ray diffraction (XRD), and thermo-gravimetric analysis (TGA) were used to analyze the structural and chemical changes in the fibers after chemical treatment. Preliminary characterization of the fiber-reinforced geopolymer using SEM-EDX and TGA combined with mechanical tests provides an impetus for future work on the development of chemically treated abaca fiber-reinforced geopolymer composites.

## 2. Materials and Method

### 2.1. Sources of Raw Materials

**Figure 1:**
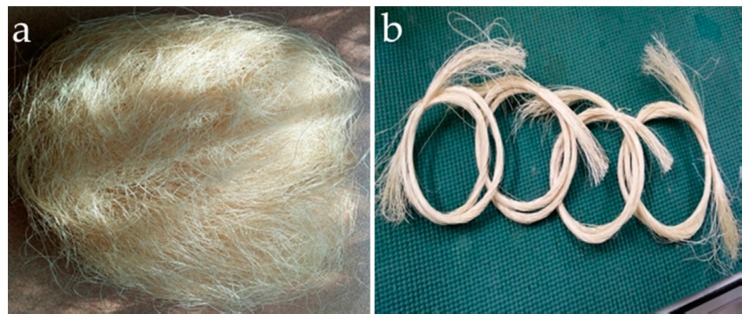
Waste abaca from Dumaguete, Philippines: (**a**) as received; and (**b**) segregated into 100 strands.

Abaca fibers are extracted by hand stripping and are subsequently segregated into standard grades based on color and texture. Residual grade abaca fibers shown in Figure 1a were collected from Dumaguete City, Philippines. The average diameter of the collected abaca strands is 162 micrometers. The fibers were cut into approximately 25 cm long and segregated to 100 strands per sample as shown in Figure 1b. The fly ash used to produce geopolymer matrix was obtained from a coal fired power plant situated at the central region of Luzon, Philippines. X-ray fluorescence (XRF, Thermo Scientific, Waltham, MA, USA) analysis of the fly ash used is summarized in Table 1. Chemicals used to treat abaca fibers, sodium hydroxide micropearls (99 wt %) and aluminum sulfate powders (99 wt %), and the water glass solution (modulus = 2.5; 34.1 wt % SiO_2_, 14.7 wt % Na_2_O) for geopolymer synthesis were supplied by a local company (Just-in-One Marketing) and were used as received.

### 2.2. Chemical Treatment of Waste Abaca Fiber

Two types of chemical treatments of abaca fibers were used in this study: (1) alkali pretreatment; and (2) aluminum sulfate treatment, which involves deposition of aluminum hydroxide on the fiber surface via precipitation. Using SAS JMP 11.1.1 software, 10 experimental runs were prescribed using Definitive Screening Design (DSD). DSD works for factor screening containing a combination of continuous and two-level categorical factors. It can estimate unbiased main effects that are completely independent of two-factor interactions and avoid confounding of any pair of quadratic effects [34,35]. Table 2 shows the factors, their levels and corresponding codes used in this design of experiment. There are three factors considered, one is a categorical factor (NaOH pretreatment) and the other two (soaking time and pH) are continuous. 

**Figure 2:**
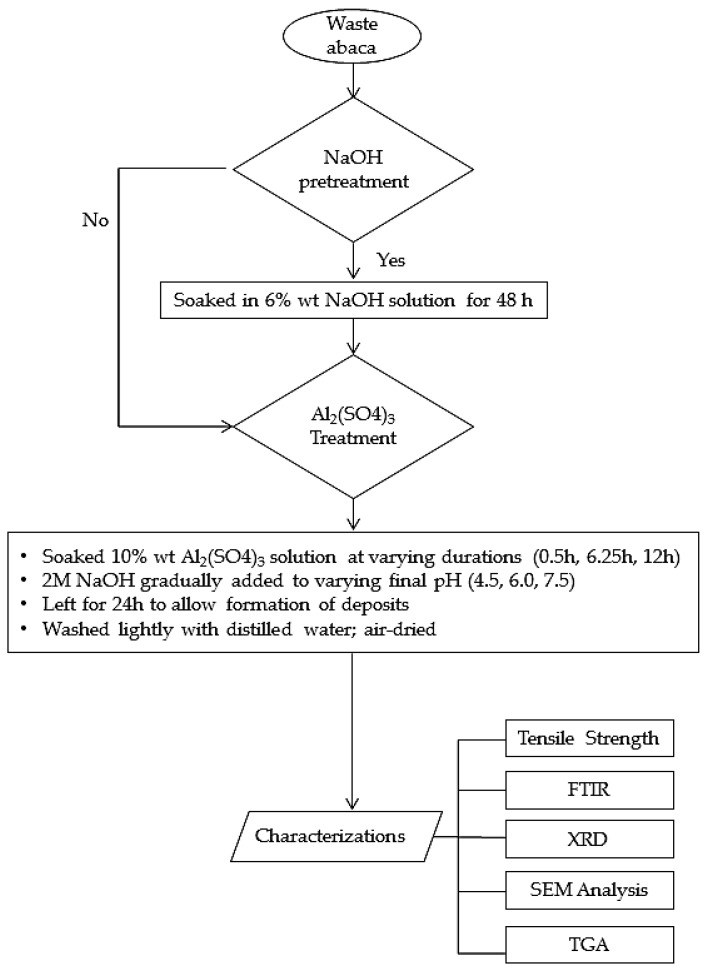
Chemical treatment and characterization of waste abaca fiber.

For the alkali pretreatment, fibers were soaked in 6% by weight NaOH solution for 48 h in a 500 mL beaker with constant agitation at room temperature. The said treatment can remove impurities of the fiber resulting to rough fiber surface [36,37]. Fibers were then removed from the NaOH solution and washed with distilled water several times. Alkali pretreated fibers were then air dried for 24 h. For the Al_2_(SO_4_)_3_ treatment, untreated and alkali pretreated fibers were soaked in 10% by weight of Al_2_(SO_4_)_3_ solution in different beakers. The initial pH of Al_2_(SO_4_)_3_ solution is 3.5. Final pH of the abaca fiber and aqueous Al_2_(SO_4_)_3_ mixture were varied based on the solubility and precipitation of Al(OH)*x* species as function of pH [38]. Soaking period was also varied to three levels, 30 min, 6.25 h and 12 h, to allow the penetration of ions into the fiber. Then, the pH was adjusted to a desired level (4.5, 6.0 or 7.5) by gradually adding 2 M NaOH solution. Subsequently, the fibers were left soaked in the mixture for another 24 h to allow further precipitation of Al(OH)_3_ on the fiber surface. Samples were then drawn from the mixture, washed with distilled water and air-dried for at least 24 h before being stored in sealed plastics. Figure 2 illustrates the flow of the chemical treatment procedure and characterization performed for the treated fiber.

### 2.3. Preparation of Abaca Fiber-Reinforced Geopolymer

Fly ash and abaca fibers (99:1 by weight) were dry-mixed to ensure uniform fiber distribution. For every 1 kg of fly ash–fiber mixture, 400 g of alkaline activator (well-mixed 80% water glass solution, 20% 12 M NaOH by weight) was added gradually while mixing using a laboratory motorized mixer. The mass ratio was chosen to achieve a geopolymer mix with considerable compressive strength and good workability [39]. Mixing was continued for about 15 min until the mixture appeared homogenous. About 30 mL of distilled water was added to attain the desired consistency. The mixture was then poured in 5 cm × 5 cm × 5 cm cube molds (for compressive strength test) and 5 cm × 5 cm × 18 cm molds (for flexural strength test), and placed on a shaker for 1 min to remove trapped bubbles. After 24 h, samples were demolded and cured in the oven at 75 °C for 24 h and then cured at ambient conditions for 28 days. A specimen of pristine geopolymer, i.e., with no abaca fiber reinforcement was also prepared to serve as the reference material. The specimens were then tested for compressive strength and flexural strength. 

### 2.4. Tensile Strength Test of Abaca Fibers

Average tensile strengths of the abaca fibers (untreated and treated) were determined by taking five strands from each bundle of 100 strands randomly. Each strand was cut to 5 cm then both ends were mounted to cardboard (2 cm × 2 cm) using quick-dry cyanoacrylate-based adhesive. Then, the prepared specimen was held by the universal testing machine (Instron model 3324, Instron, Norwood, MA, USA) with gauge length of 3 cm. The cardboard and the hardened adhesive layer in contact with the fiber protect the fiber from possible deformation due to clamping pressure. The universal testing machine (UTM) applies tensile load on the fiber strand equivalent to an extension rate of 5 mm per min and recording the stress until it breaks. Prior to fracture, each strand was placed under an optical microscope (Amscope microscope, AmScope, CA, USA) linked to a computer to measure each of their diameters. Fiber strands were assumed to have approximately circular cross-sectional areas and were computed accordingly. Tensile strength of the fiber is defined as the maximum stress it can withstand, and is computed by dividing the load at failure by the original cross-sectional area per fiber. The five readings were averaged to obtain the final value recorded.

### 2.5. Scanning Electron Microscopy—Energy Dispersive Spectroscopy (SEM-EDS) Measurements

Surface morphology and elemental microanalyses of samples were performed using Field-emission scanning electron microscope (FESEM Dual Beam Helios Nanolab 600i, FEI, Hillsboro, OR, USA) equipped with electron-dispersive X-ray spectroscopy (EDS, FEI, Hillsboro, OR, USA). The accelerating voltage and beam current for SEM analysis are 2.0 kV and 43 pA, respectively. For EDS analysis, the accelerating voltage and beam current are 15.0 kV and 0.69 nA, respectively. 

### 2.6. X-ray Diffraction (XRD) Measurements

**Figure 3:**
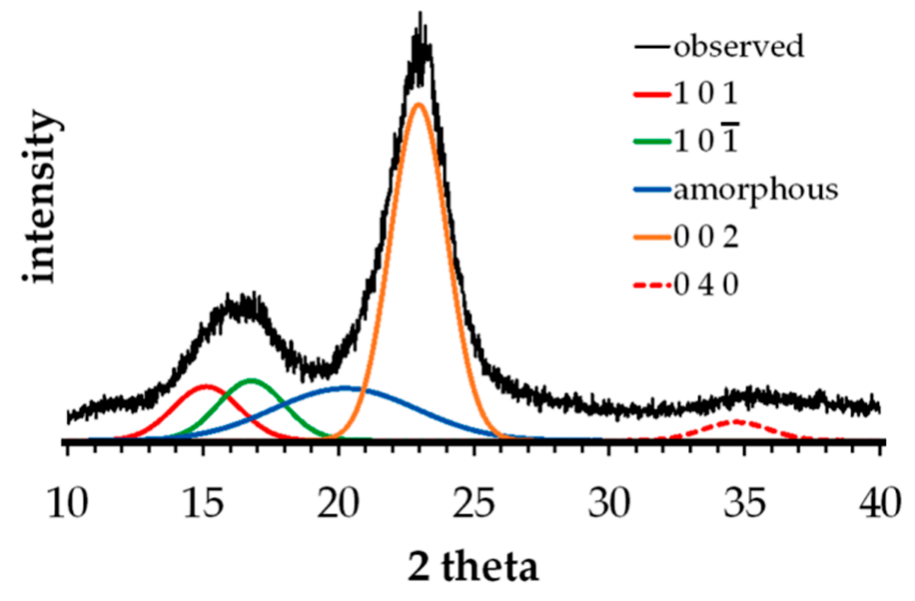
X-ray diffraction (XRD) peak deconvolution method to measure crystallinity index.

XRD (Maxima XRD-7000 Shimadzu, Tokyo, Japan) analyses were carried out using X-ray beams generated from a Cu Kα radiation source (40 kV; 30 mA) with wavelength of 1.54 Å to scan the samples from 3.00° to 70.00° 2-theta angles. The resolution for this analysis was set at 0.020, with scan speed maintained at 2° per min. The fiber’s crystallinity index (*CI*) was calculated by XRD peak deconvolution method [40]. In this method, a curve-fitting program (e.g., PeakFit v4.12) was used to identify and separate the different overlapping diffraction spectrum of both the crystalline and amorphous contributions, and then the area was calculated under each fitted Gaussian peaks. It is assumed that the observed peak broadening in the spectra is mainly attributed to the increased amorphous content in the sample. For example, four crystalline peaks (101, 10$\bar{1}$, 002 and 040) and broad amorphous peaks between 18° to 22° were identified [41] and assumed as Gaussian functions as shown in Figure 3. These Gaussian peaks were extracted from the XRD spectrum through the curve-fitting process with at least 5000 iterations, which corresponds to a coefficient of determination (r^2^) of 0.98. Based from the results of curve fitting, *CI* is calculated as the ratio of the sum of areas of all crystalline peaks to the sum of areas of all peaks (crystalline and amorphous) as shown in Equation (1) [40].

(1) $$CI=\frac{\sum A_{crystalline}}{\sum A_{crystalline}+A_{amorphous}}$$

### 2.7. Fourier Transform Infrared (FTIR) Spectroscopy Measurements

Attenuated total reflectance Fourier transform infrared (ATR-FTIR) spectroscopy (Perkin Elmer, Waltham, MA, USA) was carried out to qualitatively identify the constituents of untreated and untreated abaca fibers. Test results were obtained using Perkin Elmer FTIR Spectrometer Frontier with ATR accessory in the range of 4000–650 cm^−1^. The ATR cell is equipped with a trapezoidal diamond crystal as the internal reflection element. Baseline correction was applied to the spectrum to improve its quality without distorting the band intensities in the final spectrum.

### 2.8. Thermogravimetric Analysis (TGA) Measurements

Thermal analysis was performed using a thermogravimetric analyzer (TA Instruments TGA Q50, New Castle, DE, USA). Samples were cut into small pieces (around 5 mm) of 20–25 mg and were placed on a platinum pan, then heated from 30 to 900 °C at a rate of 10 °C per min under Argon gas at 40 mL per min purge flow. Calculations of percentage mass loss and derivative plots were done using the TA Universal Analysis software.

### 2.9. Compressive Strength and Flexural Strength Test of Geopolymer Composites

For compressive strength testing, cured cubic (5 cm × 5 cm × 5 cm) samples were subjected to a constant stress rate of 0.25 MPa/s until failure. The compressive strength (*CS*) of a sample was calculated using Equation (2):

(2) $$CS=\frac{P_{\max}}{A}$$

where *P*_max_ is the total load on the sample at failure and *A* is the calculated area of the bearing surface of the specimen.

**Figure 4:**
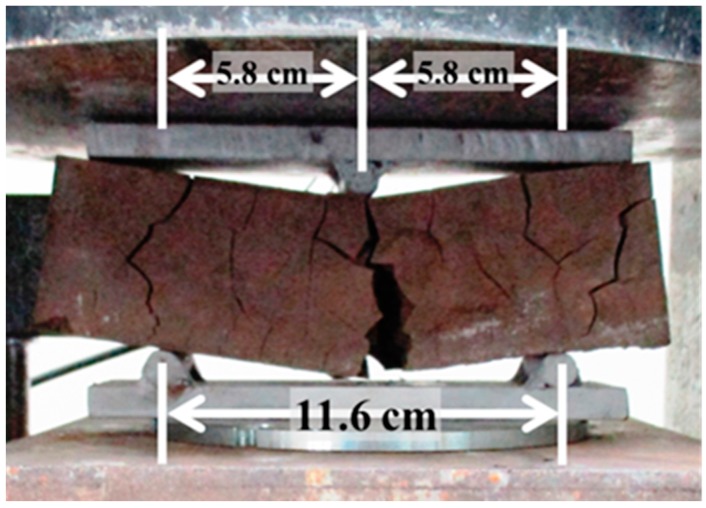
Three-point flexural strength test.

For flexural strengths or modulus of rupture (*MOR*) measurement, samples were subjected to a constant stress rate of 0.25 MPa/s using a three-point flexure loading until failure as shown in Figure 4. *MOR* was calculated based on the load at failure, cross sectional area of the sample and distance of supports using Equation (3):

(3) $$MOR=\frac{3FL}{2bd^{2}}$$

where *F* is load at failure, *L* is the distance between support or span, *b* is the width and *d* is the thickness of the specimen being tested.

## 3. Results and Discussion

### 3.1. Tensile Strength of Chemically Treated Abaca Fiber

Table 3 summarizes the measured tensile strength of 10 chemically treated waste abaca fibers using the treatment combinations according to the designed experiment from DSD (see Section 2.2). Tensile strength of treated abaca fiber in this study was observed to range from 210 to 450 MPa. Compared to the average tensile strength of untreated abaca fiber (500 MPa), all treated fibers have tensile strength lower than that of the untreated. The sample with the lowest tensile strength (210 MPa) was alkali pre-treated, soaking in Al(OH)_3_ solution for 12 h and partially neutralized up to 4.5 pH while the sample with the highest measured tensile strength (450 MPa) did not undergo alkali pretreatment but was directly soaked in Al(OH)_3_ solution also for 12 h with final pH of 6. Thus, the tensile strength of the fiber is observed to be significantly affected by the nature of chemical treatment.

**Figure 5:**
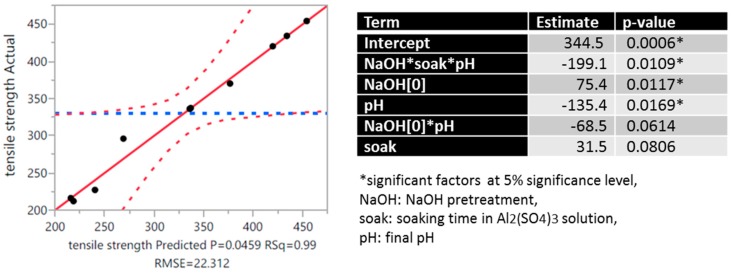
Actual vs. Predicted Plot (**- - - -** 0.05 significance curve; **- - - -**mean) for the regression model and the model coefficients.

To quantify the effect, regression analysis was conducted to model the response variable (i.e., the tensile strength) as a function of these factors namely the alkali pretreatment, soaking time and final pH. Results from the statistical analysis are summarized in Figure 5. At 5% significance level, the significant main-effect factors to tensile strength were the presence of NaOH pretreatment and the final pH at which the Al_2_(SO_4_)_3_-treated samples were neutralized (*p*-value = 0.0117 and 0.0169, respectively). NaOH pretreatment and higher final pH were generally observed to cause a decline in tensile strength. The soaking time of fiber samples in the aluminum salt solution was found to have a relatively insignificant effect to the tensile strength (*p*-value = 0.0806). Nonetheless, the interaction of the soaking time with the other two factors was found to affect the tensile strength most significantly (*p*-value = 0.0109). Furthermore, the model was able to explain 98.7% of the observed variation in tensile strength due to these factors. 

**Figure 6:**
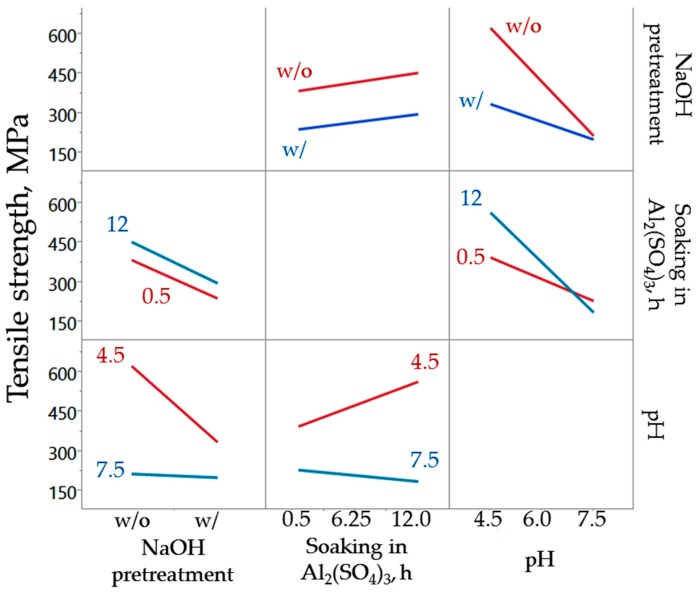
Interaction plot matrix of the factors for chemical treatment of the abaca fibers.

Figure 6 shows a matrix of interaction plots between the factors considered in the chemical treatment. Each cell of the matrix shows the interaction of the row effect with the column effect. For example, the left-most-lowest cell represents the interaction of pH (row) and NaOH pretreatment (column) effects. A line segment is plotted for each level of row effect using response values (tensile strength) predicted by the model. As observed in the interaction profile, abaca fiber samples that were not pretreated with NaOH showed a much less significant decline in tensile strength with increasing final pH, whereas alkali-treated fibers steeply declined in strength with increasing final pH. This could be explained by the extended contact with NaOH solution used for neutralization, resulting to further stripping of cellulosic components. It was found that when the Al_2_(SO_4_)_3_-treated abaca fibers were adjusted to lower final pH (4.5), the tensile strength improved with increased soaking time. When adjusted to a pH of 7.5, the tensile strength decreased with increasing soaking time in the aluminum salt solution. Note that the main purpose of Al_2_(SO_4_)_3_ treatment is to form deposits that could protect the fiber from the harsh environment of geopolymer matrix. It is possible that soaking in the aluminum salt solution inherently improves tensile strength but was counteracted by the addition of NaOH solution in adjusting the final pH, resulting instead to a decline in strength when excessive alkali solution is added to attain a high final pH.

### 3.2. Fourier Transform Infrared Spectroscopy (FTIR) of Raw and Alkali-Treated Abaca Fiber

**Figure 7:**
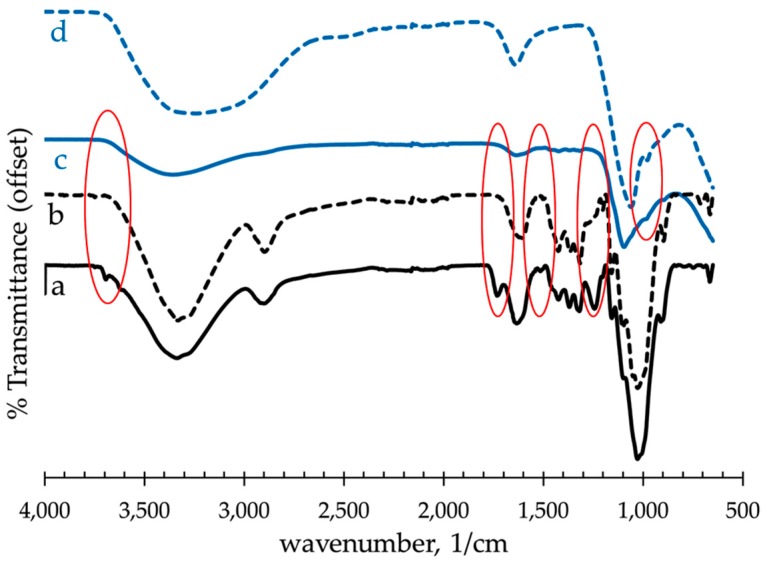
Fourier Transform Infrared Spectroscopy (FTIR) spectra of: (**a**) untreated abaca; (**b**) NaOH-treated abaca; (**c**) NaOH + Al_2_(SO_4_)_3_ treated abaca; and (**d**) precipitate or residue from the spent solution of Al_2_(SO_4_)_3_ treatment.

FTIR allows the qualitative analysis of variations in surface chemistry of natural fibers after treatment. FTIR spectra of untreated, alkali-treated and NaOH-then-Al_2_(SO_4_)_3_ treated abaca fibers are shown in Figure 7a–c. In addition, Figure 7d describes the spectra of dried precipitate or residues from the spent solution after the Al_2_(SO_4_)_3_ treatment of abaca fibers. Note that the spectra in these figures were offset vertically to observe the difference more clearly. Table 4 shows the observed bands in FTIR spectra and their assignments to functional groups. In general, untreated abaca fiber showed loss of functional groups based on the comparison of its FTIR spectrum to that of the NaOH-treated fibers. Peaks that receded in the treated fibers over frequencies 1510 cm^−1^, 1430 cm^−1^ and 1250 cm^−1^ could entail loss of phenolic groups, signifying removal of large amount of lignin and pectin whose structure is a phenolic polymer. Another visible difference was observed at 1740 cm^−1^ due to C=O stretching vibration which is a characteristic band of hemicellulose [42]. The disappearance of this band indicates the dissolution of hemicellulose after the alkali pretreatment. However there is no evidence indicating complete removal of these impurities. The decrease in tensile strength of the samples could be attributed to these extractions. In addition, characteristic peak of Al(OH)_3_ at 990 cm^−^^1^ corresponding to Al–O bonds [43] was observed in the spectra of NaOH-then-Al_2_(SO_4_)_3_ treated abaca fiber, and the precipitate or residues from the spent solution. Thus, the FTIR results confirm the possible formation of Al(OH)_3_ during neutralization and its deposition on the abaca surface.

### 3.3. XRD Analysis of Untreated and Alkali-Treated Abaca Fibers

**Figure 8:**
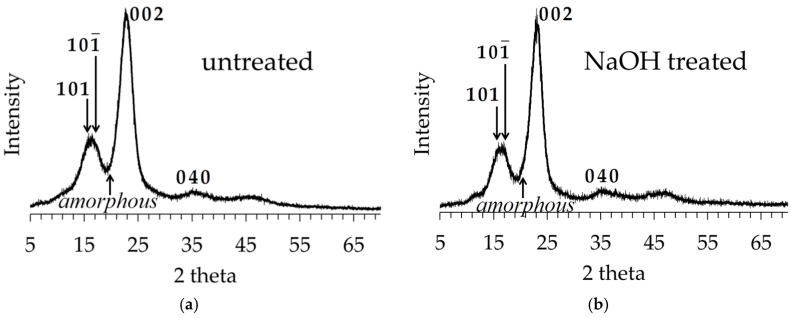
XRD spectra of untreated (**a**) and NaOH-treated (**b**) abaca fibers.

Figure 8 shows the X-ray spectra or diffractograms of untreated and NaOH-treated abaca fibers. Note that the first two crystalline peaks cannot be observed separately because they overlapped. Moreover, due to the very broad XRD spectrum of the amorphous cellulose, it overlapped with the first three crystalline peaks (101, 10$\bar{1}$, and 002). In this figure, one can notice the broader peak around the peak 002 of untreated fiber as compared with that of treated fiber. Accordingly, fitted Gaussian crystalline peaks were obtained using the XRD peak deconvolution method described in Section 2.6 to quantify the individual contributions of these components to the overall XRD spectrum of the fiber sample. This was then used to compute the relative crystallinity values to interpret the changes in the cellulose-based structure of the fiber after treatment. Results from the Gaussian peak fitting showed the presence of the four major crystalline peaks of cellulose (101, 10$\bar{1}$, 002 and 040) which can be observed at the following 2-theta angles, 15.1°, 16.8°, 22.9° and 34.7°, respectively. The broad peak for the amorphous cellulose was observed at 2-theta of 20.2°. Thus, the calculated crystallinity indices (CI) from the XRD peak deconvolution method using Equation (1) for the untreated and NaOH-treated abaca fibers were found to be 61.0% and 78.8%, respectively. It is anticipated that after sufficient alkali pretreatment, the fiber crystallinity index is bound to increase due to the extraction or removal of its amorphous components including hemicelluloses, lignin and other non-cellulosic components which allow the cellulosic fibers to adopt a more crystalline structure [31]. The observed increase in crystallinity due to the removal of lignin and hemicellulose is also in agreement with the results of FTIR analysis. 

### 3.4. Morphology and Elemental Microanalysis of Untreated and Treated Abaca Fiber Surface through SEM-EDS

**Figure 9:**
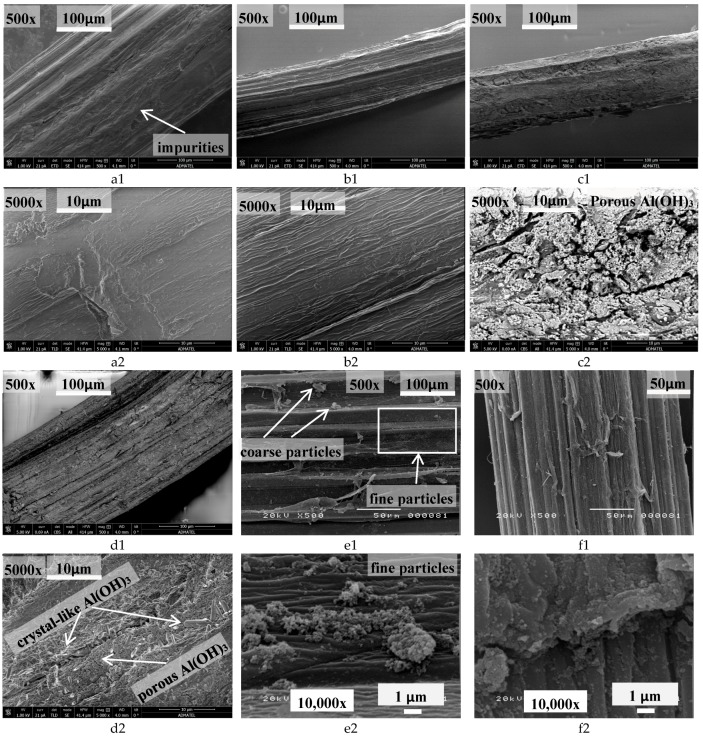
Scanning electron microscopy (SEM) images at low and high magnification of raw (untreated), NaOH-treated and Al_2_(SO_4_)_3_-treated abaca fibers: (**a**) raw (untreated); (**b**) NaOH treated; (**c**) without NaOH pretreatment; 12 h soaking; final pH = 6; (**d**) with NaOH pretreatment; 12 h; pH = 6; (**e**) without NaOH pretreatment; 12 h; pH = 4.5; and (**f**) with NaOH pretreatment; 0.5 h, pH = 6. Low and high magnification are labeled as “1” and “2”, respectively.

Effect of alkali pretreatment to the morphology and chemistry of fiber surface is important in the development fiber–matrix interaction. SEM-EDS can also provide evidences of the surface modification in terms of morphological and elemental microanalyses. Based on the results of FTIR analysis, there are fiber components from the untreated fiber that were removed after its contact with NaOH solution, thus it is expected to observe difference with their morphology. Figure 9 shows the SEM images of raw and treated abaca fibers. Figure 9a shows impurities on the textured surface of untreated abaca fiber. After treatment with NaOH solution, impurities were almost removed resulting to a more uniform but rougher or corrugated surface as shown in Figure 9b. This may be a result of stripping off the lignin and hemicellulose from fibers in alkali solution as indicated in the FTIR analysis. It is evident in the images that corrugation on the fiber surface became sharper after treatment. This was observed among all the five treated samples that underwent SEM analysis. Corrugation on the fiber surface indicates a better fiber–matrix interface by virtue of mechanical interlocking. Such corrugation in the surface indicates higher frictional bond between the two components, which has previously shown to be effective in resisting shear and bending stress [44].

In Figure 9c, SEM image of a treated abaca fiber (no alkali pretreatment, 12 h soaking; final pH = 6) indicates the Al(OH)_3_ deposits which almost covered the fiber surface. At higher magnification (5000×; Figure 9c1), the Al(OH)_3_ particles were observed to have porous structure. Figure 9d1 shows the SEM image of another treated fiber (NaOH pretreated, 12 h soaking; final pH = 6). Similar to the previous image, it can be observed that the fiber is almost completely covered with Al(OH)_3_ particles. At higher magnification (5000×; Figure 9d2), porous structure is still present with additional crystal like features. However, in Figure 9e,f, coarse and fine Al(OH)_3_ particles having porous and non-porous structures were observed on the treated abaca fiber covering partially the surface. This implies that various forms of Al(OH)_3_ were deposited on the surface of abaca fibers that were treated differently.

**Figure 10:**
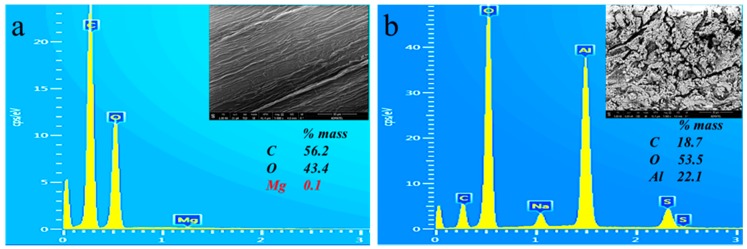
Sample EDS spectra of: (**a**) NaOH-treated; and (**b**) Al_2_(SO_4_)_3_-treated abaca.

EDS analysis was also performed on the surface of alkali treated abaca surface as shown in Figure 10. A region of interest was selected to measure the elemental composition in the said surface. However, it should be noted that the quantification error could be large as sample volume for EDS is small in an order of few micrometers in diameter. Nevertheless, the results of EDS is indicative of the presence of compound with known chemical structure. For example, in Figure 10a, the measured carbon to oxygen mass ratio (C/O) is around 1.3 whereas theoretically (based on the chemical formula) the C/O of cellulose is 0.9 and C/O of lignin is 2.9 [45,46]. This is indicative that the lignin and hemicellulose were almost removed from abaca fiber after alkali treatment. Moreover, the computed O/Al values in regions such as that of Figure 10b range from 2.8 to 3.2. This is indicative of the presence of aluminum compounds in the form of aluminum hydroxide which was confirmed from the FTIR results. Note that the said O/Al calculation is based on the assumption that all oxygen atoms detected are from the organic components of abaca fiber and aluminum hydroxide deposits only, and that the carbon to oxygen mass ratio (C/O) of fiber is 1.3 to determine the oxygen mass from the organic.

### 3.5. Thermogravimetric Analysis of Raw and Treated Abaca Fiber

Thermal stability of chemically treated abaca fibers was determined using thermogravimetric analysis (TGA). In TGA, thermal stability was studied in terms of weight losses under argon atmosphere with respect to temperature ramping (10°/min) from 30 °C to 900 °C. Note that natural fibers are mainly composed of hemicellulose, cellulose and lignin. Thermal degradation of these components was reported to take place in different temperature ranges [25,47]. The observed thermal decompositions are summarized in Table 5. The value of T_max_ represents the temperature at which the maximum decomposition rate was observed based from the differential thermogravimetry (DTG) data. 

**Figure 11:**
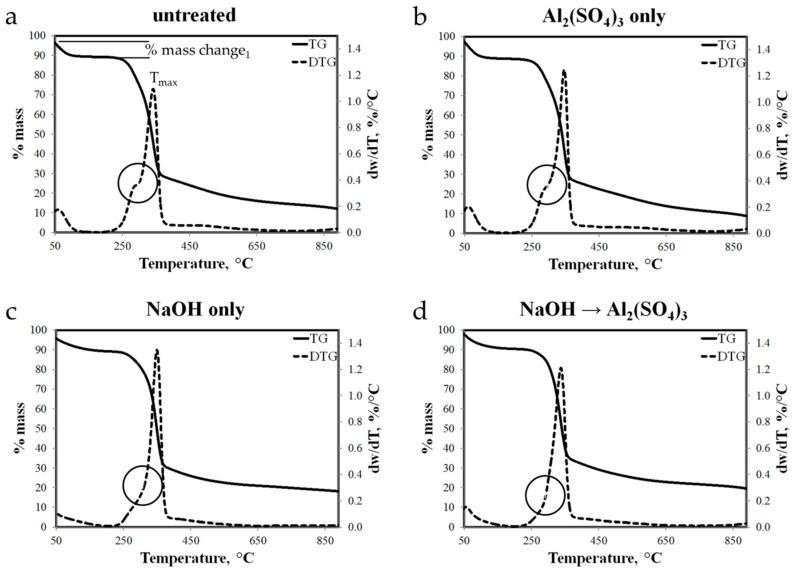
Thermograms of: (**a**) untreated; (**b**) treated solely with Al_2_(SO_4_)_3_; (**c**) alkali-pretreated; and (**d**) alkali and Al_2_(SO_4_)_3_ solution-treated abaca fiber samples.

Thermograms of abaca samples treated differently in Figure 11 show the T_max_, percentage mass loss and rate of decomposition at T_max_ for each stage of thermal decomposition. For the four abaca samples, the initial mass loss ranging from 7.8 to 10.3 percentage mass loss from 50 °C to around 120 °C is mainly due to the evaporation of free and absorbed moisture on the fiber. Considering the untreated abaca fiber, the main decomposition event was observed from 200 °C to 370 °C equivalent to percentage mass loss of 78.2%. At this temperature range, a shoulder before the peak located at 300 °C and a peak was observed at 345 °C (DTG). Fastest rate of decomposition with respect to temperature was recorded at 1.1 percentage mass loss per °C. From the onset of the main thermal decomposition to the location of shoulder, the mass loss is mainly due to the decomposition of hemicellulose. As the temperature increases, more cellulose decomposed and lignin started its degradation. Beyond 370 °C, slower decomposition of the remaining components proceeded up to 900 °C resulting to a total mass loss of 87.1% or residual mass of 12.9%. On one hand, thermogram of abaca fiber treated with Al_2_(SO_4_)_3_ only is shown in Figure 11b. The features of this plot are similar to that of untreated abaca except for the less obvious shoulder in DTG plot and lower percentage mass loss in the main decomposition event, and thus leaving more residues. These differences can be associated to the partial removal of hemicellulose and lignin during the neutralization process by adding NaOH solution. Figure 11c,d show thermograms of abaca fiber treated with NaOH, and NaOH then with Al_2_(SO_4_)_3_, respectively. It can be observed that the shoulder feature is no longer present in the DTG plots of these samples. Since these samples were exposed to a higher concentration NaOH solution for a longer period, more hemicellulose particles were removed from the fiber during the chemical treatment. 

Thermal decomposition of Al(OH)_3_ as reported from a previous study [48] starts at about 240 °C up to about 400 °C overlapping with the thermal decomposition of the organic components of abaca fibers. Furthermore, due to the small amount of Al(OH)_3_ deposited on abaca surface, the effect of Al(OH)_3_ thermal decomposition to the overall thermograms of Al_2_(SO_4_)_3_ treated fibers was not obvious. 

### 3.6. Preliminary Characterization of Fiber-Reinforced Geopolymers

**Figure 12:**
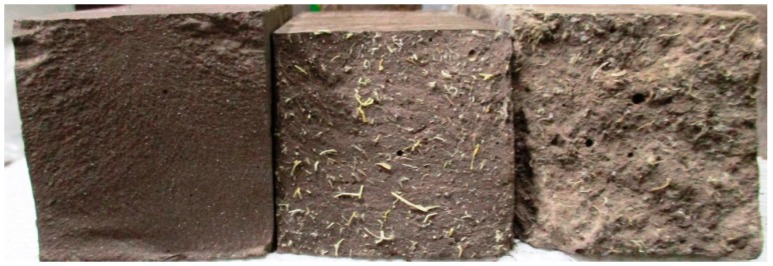
Fracture surface of pristine geopolymer, reinforced with untreated abaca and reinforced with treated abaca.

Incorporating abaca fiber (about 1% by weight of fly ash) in geopolymer matrix improved the compressive strength of untreated fiber-reinforced (25.9 MPa) and treated fiber-reinforced composite (22.2 MPa) by 20% and 3%, respectively, compared to that of a pristine geopolymer (21.6 MPa). Likewise, the flexural strength of the untreated fiber reinforced (5.5 MPa) and the fiber-reinforced composite (7.3 MPa) was also improved by 95% and 161%, respectively, compared to that of a pristine geopolymer (2.8 MPa). Such results indicate that the fiber’s main contribution is to improve primarily the composite’s flexural strength and not much of its compressive strength since the fibers control the cracking that gives rise to a “graceful” fracture by bridging across the cracks. Figure 12 shows the fracture surface of the pristine geopolymer and those of the abaca fiber-reinforced. Moreover, a significant improvement in flexural strength of the composite reinforced with chemically treated abaca fiber suggests a better interfacial adhesion between the fiber and the matrix. However, the difference of improvement between the mechanical properties of geopolymer reinforced with untreated fiber and treated fiber still needs further verification in future works.

**Figure 13:**
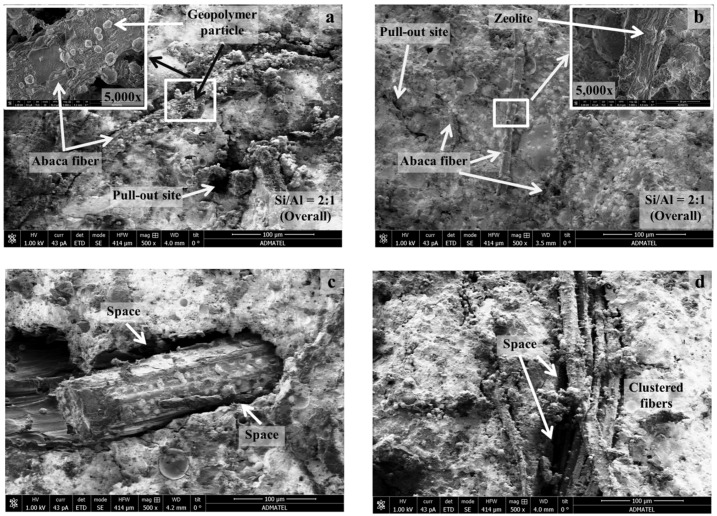
SEM images of composite fractured surfaces: (**a**) reinforced with untreated abaca; (**b**) reinforced with treated abaca; (**c**) interface between untreated abaca fiber and geopolymer matrix and (**d**) interface between treated abaca fiber and geopolymer matrix.

In this paper, we focus on the microstructure characterization of the abaca-fiber reinforced geopolymer composites through SEM and thermogravimetric analysis. Representative fractured surfaces of the composites were analyzed using SEM-EDS as shown in Figure 13. Heterogeneous geopolymer of different morphology such as textured spheres, porous and crystal-like structures were observed for both composites samples. Results of EDS indicated that Si and Al in the geopolymer have an overall atomic ratio of 2:1. Fibers were clearly embedded on the geopolymer matrix as shown from the micrographs. For composites reinforced with untreated abaca (Figure 13a,c), visible gaps were observed between the geopolymer matrix and fiber indicating poor interfacial adhesion. This is likely due to the incompatibility of the fiber and matrix. Circular-like particles were also observed on the surface, which are likely to be geopolymer precursors or nuclei that adhered on the abaca surface. On the other hand, for the composites reinforced with treated abaca fibers (Figure 13b,d), narrower gaps were observed between the matrix and treated fiber. The zeolite-like particles on the fiber surface indicate that reaction took place on the surface for these structures to form. Clustering of abaca fibers was also observed in other region of the fractured surface. Although geopolymer and zeolite deposits were observed which indicates the interaction between the geopolymer matrix and fiber surface, pull out sites were observed suggesting the formed interfacial adhesion was not enough in some regions. Nevertheless, the pulling out of these fibers during the loading process absorbs energy that results to the improvement of the flexural strength of composite.

**Figure 14:**
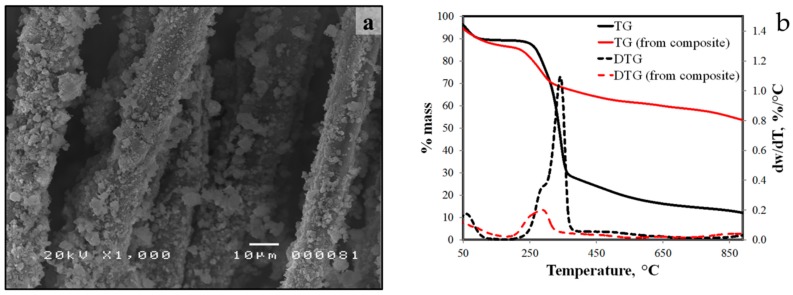
SEM image (**a**) and thermogram of untreated abaca fibers and treated abaca fibers (**b**) from the geopolymer composite.

After compressive strength testing, treated abaca fibers from the fractured composites were removed from the composite and subjected to TG analysis using the same method of thermal stability analysis of free abaca fibers. Thermogram of abaca fibers collected from the composite is shown in Figure 14b. T_max_ of the main thermal decompositions was observed at 287 °C and the total percentage mass loss was 43.3%. In comparison to free treated abaca fibers, the percentage mass loss for the main decomposition event is lower for fibers from the composite than the free fiber samples. Moreover, the measured rate of decomposition at T_max_ is lower at 0.2% per °C versus the rate at T_max_ of free abaca fiber at average of 1.2% per °C. This is likely due to sites of geopolymer growth formed on the surface of the treated fibers. The abaca fibers were covered with geopolymer particles and thus protecting the fibers from thermal degradation.

## 4. Conclusions

Chemical treatment of waste abaca fibers modifies their structure and chemical composition, with the modification depending on the treatment conditions. The high tensile strength among the treated fibers was achieved without alkali pretreatment (6% by wt NaOH solution for 48 h), soaked for 12 h in aluminum sulfate solution and neutralized to pH 6. FTIR results indicate that the alkali pretreatment successfully dissolved some of the amorphous and hydrophobic components such as lignin, pectin and hemicellulose, and the XRD results indicate an increase in fiber crystallinity that may be attributed to such dissolution. Results from thermogravimetric analysis (TGA) confirm the removal of lignin, pectin and hemicellulose from the fibers, and also suggest that the geopolymer itself coat the treated abaca fibers and protect them from thermal degradation. The Al_2_(SO_4_)_3_ treatment is effective to form Al(OH)_3_ deposits that roughen the surface for better fiber- matrix interface and could also protect the fiber from the harsh environment of the geopolymer matrix. Preliminary results on abaca fiber-reinforced geopolymer indicate better adhesion for the treated fiber/matrix which results in an improved flexural strength as compared to the pristine geopolymer. Future works will further investigate the effect of chemical treatment on other properties of fiber such as swelling, as well as the mechanical properties of such fiber-reinforced geopolymer composites.

## Figures and Tables

**Table 1:** X-ray fluorescence (XRF) analysis of fly ash.

Oxide	SiO_2_	Fe_2_O_3_	CaO	Al_2_O_3_	MgO	K_2_O	Others
Mass %	33.9	26.5	13.6	13.5	7.9	1.2	3.4

**Table 2:** Factors and levels used for the chemical treatment.

Factor/Level (Coded)	−1	0	+1
NaOH pretreatment	-	No	Yes
Soaking time in Al_2_(SO_4_)_3_, hrs	0.5	6.25	12
Final pH	4.5	6.0	7.5

**Table 3:** Measured response (tensile strength) at different treatment conditions.

NaOH Pretreatment	Soaking Time (h)	Final pH	Tensile Strength (MPa)
No	12	6.0	450 ± 160
No	0.5	7.5	430 ± 130
No	6.25	6.0	420 ± 140
Yes	12	7.5	370 ± 82
No	0.5	4.5	340 ± 97
Yes	6.25	4.5	340 ± 140
Yes	6.25	6.0	300 ± 82
Yes	0.5	6.0	230 ± 82
No	6.25	7.5	220 ± 69
Yes	12	4.5	210 ± 83

**Table 4:** Functional Groups of Observed Bands in FTIR spectra of abaca fiber [25,42,43].

Wavenumber (cm^−1^)	Vibration	Source
3690	Free OH	moisture
3350	O–H linked stretching	Polysaccharide
2920	C–H stretching	Cellulose, Hemicellulose
1740	C=O stretching	Hemicellulose
1630	OH in H_2_O, bending	Moisture
1510	C=C aromatic symmetrical stretching	Lignin
1430	CH_2_ symmetric bending	Pectin, lignin
1250	C–O aryl group	Lignin
980	Al–O stretching	Al(OH)_3_
900	Glycosidic bonds symmetric ring stretching	Polysaccharide

**Table 5:** Decomposition Temperature of Fiber Components [25,47].

Components	Temperature of Decomposition, °C	T_max_ Based on DTG, °C
Moisture	30–100	80
Hemicellulose (Xylan)	160–350	245 (side chain), 298 (backbone)
Cellulose	240–365	335
Lignin	300–500	337
